# Depression and Its Association With Microvascular and Macrovascular Complications in Type 2 Diabetes

**DOI:** 10.1155/jdr/2234488

**Published:** 2026-05-11

**Authors:** Mariam Wehbi, Ahmad Al-Bitar, Zaynab Alourfi, Younes Kabalan

**Affiliations:** ^1^ Internal Medicine-Endocrinology and Metabolism Department, Faculty of Medicine, Damascus University, Damascus, Syria, damascusuniversity.edu.sy; ^2^ Faculty of Medicine, Damascus University, Damascus, Syria, damascusuniversity.edu.sy; ^3^ Endocrinology and Metabolism Department, National University Hospital, Faculty of Medicine, Damascus University, Damascus, Syria, damascusuniversity.edu.sy

**Keywords:** depression, diabetic complications, glycemic control, microvascular complications, Type 2 diabetes mellitus

## Abstract

**Background:**

Type 2 diabetes mellitus (T2DM) and depression frequently co‐occur, but the specific clinical factors driving this association remain incompletely understood, particularly in Middle Eastern populations.

**Objective:**

This study is aimed at investigating the association between T2DM and depression and identifying which clinical factors (complications, glycemic control, and treatment modality) are most strongly associated with depression risk in a Syrian patient cohort.

**Methods:**

A case‐control study enrolled 225 patients with T2DM and 226 nondiabetic controls. Depression was assessed using the Hamilton Depression Rating Scale (HDRS) and the Depression, Anxiety, and Stress Scales (DASS‐21). Associations were analyzed using chi‐square tests and binary logistic regression, with adjustment for age and sex in subgroup analyses.

**Results:**

Depression prevalence was significantly higher in the T2DM group (68.4% by HDRS and 68.9% by DASS‐21) than in controls (40.3% and 41.6%, respectively). A T2DM diagnosis was associated with approximately threefold increased odds of depression (OR = 3.22, 95% CI [2.19–4.74] for HDRS; OR = 3.12, 95% CI [2.11–4.58] for DASS‐21). Microvascular complications were associated with a marked increase in depression odds (OR = 17.05 − 17.89). Achieving glycemic control was strongly protective (OR = 0.116 − 0.125). Complex insulin‐based regimens were associated with greater depression severity, even when the binary presence of depression did not differ significantly by treatment type.

**Conclusion:**

In this Syrian cohort, T2DM was associated with a threefold higher odds of depression, but this risk was not uniform. The presence of microvascular complications was the strongest associated factor. These findings support the implementation of routine depression screening in diabetes care, particularly for patients with complications or complex treatment regimens, and highlight that intensive diabetes management may also contribute to depression prevention.

## 1. Introduction

Type 2 diabetes mellitus (T2DM) is associated with a substantially elevated prevalence of depression. Contemporary meta‐analyses indicate that major depressive disorder is approximately twice as common among individuals with T2DM compared to the general population, and this comorbidity predicts worse glycemic outcomes, lower quality of life, and increased mortality [1, 2]. The co‐occurrence of these conditions complicates clinical management and amplifies the overall disease burden.

The relationship between T2DM and depression is recognized as bidirectional. Depression increases the risk of incident T2DM through behavioral (e.g., poor diet and physical inactivity) and biological pathways (e.g., chronic low‐grade inflammation and hypothalamic–pituitary–adrenal axis dysregulation). Conversely, the psychological demands of managing a chronic illness—including treatment adherence, lifestyle modifications, and fear of complications—commonly precipitate or worsen depressive episodes in those already diagnosed [3, 4]. Recent mechanistic research has identified shared pathways including chronic inflammation, gut–brain axis dysregulation, and metabolic abnormalities [5].

While the general association is well established, current research seeks to identify the specific clinical factors that mediate depression risk within the T2DM population. Accumulating evidence suggests that the psychological burden is not a uniform consequence of the diagnosis itself but is tightly correlated with disease severity and progression. A 2025 meta‐analysis found a substantial prevalence of depression directly linked to the presence of microvascular complications such as neuropathy and retinopathy, suggesting that tangible, often debilitating physical symptoms are potent contributors to psychological distress [6]. Similarly, poor glycemic control has been robustly associated with higher depression prevalence, indicative of a vicious cycle in which depression impairs self‐care, which in turn worsens glycemic control and deepens depressive symptoms [7, 8].

Most existing studies have been conducted in high‐income Western populations, and limited data are available from the Middle East and North Africa region, where healthcare infrastructure, cultural attitudes toward mental health, and diabetes epidemiology may differ substantially. A recent study from India reported a depression prevalence of only 6.2% among T2DM patients using the PHQ‐9 [9], highlighting the need for region‐specific data. Furthermore, few studies have simultaneously quantified the independent contributions of microvascular complications, macrovascular complications, glycemic control, and treatment modality to depression risk within a single cohort using two validated assessment instruments.

Therefore, this study was designed to investigate the association between T2DM and depression in a Syrian patient cohort and to identify which specific clinical factors are most strongly associated with depression risk. We hypothesized that the prevalence and severity of depression would be more strongly associated with the clinical burden of diabetes—particularly the presence of long‐term complications, poor glycemic control, and intensive treatment regimens—than with the diagnosis of diabetes alone. To our knowledge, this is the first study in a Syrian cohort to quantify the independent contributions of these factors using both a clinician‐administered scale (Hamilton Depression Rating Scale [HDRS]) and a self‐report instrument (Depression, Anxiety, and Stress Scales‐21 [DASS‐21]). Given the cross‐sectional design, we describe associations rather than causal pathways.

## 2. Methodology

### 2.1. Study Design and Population

This case‐control study was designed to compare individuals with T2DM against nondiabetic controls to identify clinical factors associated with depression. The study followed the Strengthening the Reporting of Observational Studies in Epidemiology (STROBE) guidelines for case‐control studies. A completed STROBE checklist is provided as a separate file.

The sample size was calculated using G∗Power software (Version 3.1). Based on prior literature [10], we assumed a prevalence of depression of approximately 25% in the control group and an odds ratio (OR) of 2.0 for depression in T2DM compared to controls. With an alpha error of 0.05 and a power of 80%, the minimum required sample size was estimated to be 200 per group. We recruited 225 participants per group to account for potential incomplete data.

The case group consisted of patients diagnosed with T2DM, recruited consecutively from the outpatient endocrinology clinics at Al‐Mouwasat University Hospital and the National University Hospital during routine follow‐up visits between April 2024 and April 2025. The control group comprised apparently healthy adults without a known diagnosis of diabetes, recruited from the community using convenience sampling (hospital staff, visitors, and individuals from local community centers who responded to study advertisements). Cases and controls were frequency‐matched on age categories (10‐year bands) and sex.

### 2.2. Participant Selection

Inclusion criteria for the case group are as follows: confirmed diagnosis of T2DM. Inclusion criteria for the control group are as follows: apparently healthy adults without a prior diabetes diagnosis. All potential controls underwent screening with a fasting plasma glucose (FPG) test; individuals with FPG ≥ 126 mg/dL (7.0 mmol/L) were excluded. An oral glucose tolerance test (OGTT) was not performed; this limitation is acknowledged.

Exclusion criteria for both groups are as follows: pregnancy, current diabetic ketoacidosis (for cases), Type 1 diabetes mellitus, pre‐existing psychiatric disorders (e.g., bipolar disorder and schizophrenia), current use of medications with known psychotropic effects (e.g., antipsychotics and antidepressants), or other medical conditions associated with depression (thyroid disorders, adrenal diseases, and malignancies).

### 2.3. Data Collection and Procedures

Data were collected using a structured, study‐specific Case Report Form (CRF) piloted prior to the main study. Demographic information and medical history were obtained via patient interview. For the T2DM group, disease duration, medication regimens, and known complications were recorded and verified against medical records where available. Blood samples were collected from all participants for laboratory analysis of fasting blood glucose, glycated hemoglobin (HbA1c), creatinine, and hemoglobin, processed at the hospital′s central laboratory.

Depression was assessed using two validated instruments: the clinician‐administered HDRS and the self‐reported DASS‐21. The HDRS was administered by trained study physicians; a total score ≥ 8 indicated clinically significant depressive symptoms (*mild*: 8–16, *moderate*: 17–23, and *severe*: ≥ 24) [8]. The DASS‐21 was self‐administered; a depression subscale score ≥ 10 indicated clinically significant symptoms (*mild*: 10–13, *moderate*: 14–20, *severe*: 21–27, and *extremely severe*: ≥ 28) [9]. Both instruments were used in their validated Arabic versions [11, 12].

For T2DM patients, a systematic assessment of diabetic complications was conducted:

• *Acute complications* (past year): hypoglycemia requiring assistance (documented from medical records or patient report of severe episode requiring third‐party help) and diabetic ketoacidosis (documented from medical records).
• *Chronic complications*: ischemic heart disease (symptoms plus ECG findings), cerebrovascular disease (history of stroke or transient ischemic attack), ocular complications (mandatory ophthalmology consultation), peripheral neuropathy (symptoms of numbness, burning pain, or monofilament testing where available), diabetic foot ulcers (clinical examination), and chronic kidney disease (estimated glomerular filtration rate < 60 mL/min/1.73 m^2^ on two occasions).

Control participants underwent eligibility screening with FPG. After providing informed consent, they completed the CRF via interview and the DASS‐21. A study physician, blinded to DASS‐21 results, then conducted a brief medical interview and administered the HDRS.

### 2.4. Definition of Variables and Statistical Analysis

Glycemic control was defined as “controlled” if HbA1c ≤ 7*%* or, in the absence of HbA1c, fasting blood glucose ≤ 130 mg/dL. The use of fasting blood glucose as a fallback may overestimate glycemic control compared to HbA1c; this limitation is acknowledged.

Data were analyzed using SPSS Version 25 and Microsoft Excel. Descriptive statistics are as follows: frequencies and percentages for categorical variables and means and standard deviations for continuous variables. Inferential statistics are as follows: chi‐square tests for associations between categorical variables. Binary logistic regression was used to calculate ORs with 95% confidence intervals (CIs). The primary regression models were unadjusted because cases and controls were frequency‐matched on age and sex; additional models adjusting for age, sex, and education are reported in Table S1. For analyses within the T2DM group (complications, glycemic control, and treatment modality), models were adjusted for age and sex. A *p* value < 0.05 was considered statistically significant.

For depression severity by treatment modality, chi‐square tests were performed: DASS‐21: *χ* ^2^(2) = 32.14, *p* < 0.001; HDRS: *χ* ^2^(2) = 68.50, *p* < 0.001. Exact test statistics and degrees of freedom are reported in the Results section.

### 2.5. Ethical Considerations

This study received approval from the Biomedical Research Ethics Committee (BMREC) at Damascus University (ID Number: MD‐024011‐4150). All participants provided written informed consent after receiving a complete description of the study protocol. Data were anonymized and maintained confidentially.

### 2.6. Data Availability Statement

The authors confirm that the data supporting the findings of this study are available within the article and its supporting information. Additional anonymized data may be requested from the corresponding author.

## 3. Results

### 3.1. Participant Characteristics

The study cohort comprised 451 participants: 225 individuals with T2DM (49.9%) and 226 nondiabetic controls (50.1%). Baseline demographic and clinical characteristics are presented in Table 1. The mean age of all participants was 44.3 ± 14.4 years (range 19–72 years). The cohort was predominantly female (67.0%) and married (76.5%), with a majority employed (66.5%).

**Table 1:** Baseline demographic and clinical characteristics by study group.

Characteristic	Category	T2DM group (*n* = 225)	Control group (*n* = 226)	*p* value
Age (years), mean ± SD		45.1 ± 13.8	43.5 ± 15.0	0.235
Sex, *n* (%)	Female	163 (72.4)	139 (61.5)	0.015
	Male	62 (27.6)	87 (38.5)	
Marital status, *n* (%)	Single	46 (20.4)	57 (25.2)	0.365
	Married	176 (78.2)	169 (74.8)	
	Divorced	3 (1.3)	0 (0.0)	
Educational attainment, *n* (%)	Illiterate	25 (11.1)	17 (7.5)	< 0.001
	Primary	52 (23.1)	30 (13.3)	
	Preparatory	32 (14.2)	21 (9.3)	
	Secondary	20 (8.9)	29 (12.8)	
	University	96 (42.7)	129 (57.1)	
Employment status, *n* (%)	Employed	144 (64.0)	156 (69.0)	0.265
	Unemployed	81 (36.0)	70 (31.0)	
Depression (HDRS ≥ 8), *n* (%)	Depressed	154 (68.4)	91 (40.3)	< 0.001
	Not depressed	71 (31.6)	135 (59.7)	
Depression (DASS − 21 ≥ 10), *n* (%)	Depressed	155 (68.9)	94 (41.6)	< 0.001
	Not depressed	70 (31.1)	132 (58.4)	

As shown in Table 1, the T2DM and control groups were comparable in age, marital status, and employment distribution. However, the T2DM group had a higher proportion of females (72.4% vs. 61.5%, *p* = 0.015) and lower educational attainment (university degree: 42.7% vs. 57.1%, *p* < 0.001).

In the total sample, the HDRS identified depressive symptoms (score ≥ 8) in 54.3% (*n* = 245) of participants, while the DASS‐21 depression subscale (score ≥ 10) indicated a prevalence of 55.2% (*n* = 249). As shown in Table 1, the prevalence was significantly higher in the T2DM group for both measures (HDRS: 68.4% vs. 40.3%, *p* < 0.001; DASS‐21: 68.9% vs. 41.6%, *p* < 0.001). All subsequent analyses present results for each scale independently to provide convergent validity.

### 3.2. Clinical Profile of Patients With T2DM

A detailed clinical characterization of the T2DM subgroup (*n* = 225) is presented in Table 2. The majority of patients were managed with oral hypoglycemic agents (OHAs) alone (79.6%, *n* = 179). Nearly three‐quarters (73.3%, *n* = 165) were classified as having uncontrolled diabetes. This high proportion reflects local healthcare challenges in achieving glycemic targets, which is further addressed in the Discussion section.

**Table 2:** Clinical characteristics of patients with Type 2 diabetes mellitus (*n* = 225).

Characteristic	Category	*n* (%)
Treatment modality	Oral hypoglycemics only	179 (79.6)
	Insulin only	25 (11.1)
	Combination (OHA + insulin)	21 (9.3)
Glycemic control^a^	Controlled	60 (26.7)
	Uncontrolled	165 (73.3)
Microvascular complications	Any microvascular	164 (72.9)
	‐ Neuropathy	117 (52.0)
	‐ Retinopathy	87 (38.7)
	‐ Nephropathy (CKD stage ≥ 3)	70 (31.1)
Macrovascular complications	Any macrovascular	63 (28.0)
	‐ Coronary artery disease	41 (18.2)
	‐ Cerebrovascular disease	15 (6.7)
	‐ Peripheral artery disease	12 (5.3)
Acute complications (past year)	Any acute	55 (24.4)
	‐ Hypoglycemia requiring assistance	50 (22.2)
	‐ Diabetic ketoacidosis	5 (2.2)

^a^Glycemic control was defined as HbA1c ≤ 7*%* or fasting blood sugar ≤ 130 mg/dL.

Microvascular complications were highly prevalent, affecting 72.9% (*n* = 164) of patients. Among these, diabetic neuropathy was the most common (52.0%), followed by retinopathy (38.7%) and nephropathy (31.1%); many patients had more than one complication. Macrovascular complications were documented in 28.0% (*n* = 63) of the cohort, with coronary artery disease being the most frequent (18.2%). Acute complications (primarily significant hypoglycemic episodes in the past year) were reported by 24.4% (*n* = 55) of the T2DM group.

### 3.3. Association Between T2DM and Depression

Inferential analysis demonstrated a strong and statistically significant association between a diagnosis of T2DM and the presence of depression. The prevalence of depression was significantly higher in the T2DM group compared to controls, consistent across both instruments. Using the DASS‐21, 68.9% of individuals with T2DM screened positive for depression versus 41.6% of controls (*χ* ^2^ = 33.97, df = 1, *p* < 0.001). Similarly, the HDRS identified depression in 68.4% of the T2DM group compared to 40.3% of controls (*χ* ^2^ = 36.08, df = 1, *p* < 0.001).

To quantify this association, binary logistic regression was performed. Table 3 presents the unadjusted ORs because cases and controls were frequency‐matched on age and sex. A diagnosis of T2DM remained a strong predictor of depression. The odds of having depression as measured by DASS‐21 were over three times higher for individuals with T2DM compared to controls (OR = 3.12, 95% CI [2.11, 4.58], *p* < 0.001). Results using the HDRS were nearly identical (OR = 3.22, 95% CI [2.19, 4.74], *p* < 0.001). Adjusted models including age, sex, and education are provided in Table S1 and yielded similar estimates.

**Table 3:** Association between Type 2 diabetes mellitus and depression (binary logistic regression, unadjusted).

Outcome measure	Predictor	Odds ratio (OR)	95% confidence interval	*p* value
Depression (DASS‐21)	T2DM diagnosis	3.12	2.11–4.58	< 0.001
Depression (HDRS)	T2DM diagnosis	3.22	2.19–4.74	< 0.001

### 3.4. Clinical Factors Associated With Depression in Patients With T2DM

To further explore factors associated with depression within the T2DM cohort, we examined diabetic complications, glycemic control, and treatment modality. All analyses within the T2DM group were adjusted for age and sex.

#### 3.4.1. Impact of Diabetic Complications

The presence of diabetic complications emerged as the strongest predictor of depression, with effect sizes substantially exceeding that of the T2DM diagnosis alone. Macrovascular complications were associated with a more than fourfold increase in the odds of depression (DASS: OR = 4.26, 95% CI [1.90, 9.55], *p* < 0.001; HDRS: OR = 4.38, 95% CI [1.95, 9.80], *p* < 0.001).

The association was markedly stronger for microvascular complications. The odds of depression were approximately 17–18 times higher in patients with microvascular complications compared to those without. Specifically, for DASS: OR = 17.89 (95% CI [8.67, 36.97], *p* < 0.001); for HDRS: OR = 17.05 (95% CI [8.29, 35.09], *p* < 0.001). The wide CIs reflect the relatively small number of patients without microvascular complications (*n* = 61); this is acknowledged as a limitation.

#### 3.4.2. Role of Glycemic Control

A strong inverse relationship was observed between glycemic control and depression. With uncontrolled diabetes as the reference category, achieving glycemic control was significantly protective. Patients with controlled diabetes had an 87.5% reduction in the odds of depression as measured by DASS (OR = 0.125, 95% CI [0.06, 0.24], *p* < 0.001) and an 88.4% reduction as measured by HDRS (OR = 0.116, 95% CI [0.06, 0.23], *p* < 0.001).

#### 3.4.3. Influence of Treatment Modality

When considering the presence or absence of depression (binary outcome), the association with treatment type was ambiguous, reaching statistical significance with the HDRS (*p* = 0.049) but not the DASS (*p* = 0.080). However, a clearer pattern emerged when examining depression severity.

##### 3.4.3.1. Depression Severity and Treatment Modality.

A detailed analysis revealed a highly significant association between treatment regimen complexity and depression severity. For DASS‐21 (Table 4), the likelihood ratio test was significant (*χ* ^2^ = 32.14, df = 2, *p* < 0.001). Patients with mild depression were overwhelmingly managed with oral hypoglycemics only (92.6%), whereas patients with moderate‐to‐severe depression were distributed across OHAs only (50.8%), insulin only (24.6%), and combination therapy (24.6%).

**Table 4:** Distribution of T2DM patients by depression severity (DASS‐21) and treatment modality (*n* = 155) (*χ* ^2^ = 32.14, df = 2, *p* < 0.001).

Treatment modality	Mild depression (*n* = 94)	Moderate‐to‐severe depression (*n* = 61)
Oral hypoglycemics only	87 (92.6%)	31 (50.8%)
Insulin only	7 (7.4%)	15 (24.6%)
Combination (OHA + insulin)	0 (0.0%)	15 (24.6%)

For HDRS (Table 5), the association was even more pronounced (*χ* ^2^ = 68.50, df = 2, *p* < 0.001). Patients with mild depression were almost exclusively on single‐agent therapy (84.3% OHAs and 15.7% insulin). In contrast, the majority of patients with moderate‐to‐severe depression (75.0%) were on combination therapy.

**Table 5:** Distribution of T2DM patients by depression severity (HDRS) and treatment modality (*n* = 154) (*χ* ^2^ = 68.50, df = 2, *p* < 0.001).

Treatment modality	Mild depression (*n* = 134)	Moderate‐to‐severe depression (*n* = 20)
Oral hypoglycemics only	113 (84.3%)	4 (20.0%)
Insulin only	21 (15.7%)	1 (5.0%)
Combination (OHA + insulin)	0 (0.0%)	15 (75.0%)

## 4. Discussion

In this case‐control study of 451 participants (225 with T2DM and 226 nondiabetic controls), we observed a significant association between T2DM and depression. The prevalence of depression was approximately 68% in the T2DM group compared to 41% in controls, corresponding to an OR of approximately 3.2. These findings are consistent with the established literature but extend current knowledge by quantifying the independent contributions of specific clinical factors within a Syrian cohort.

The most notable finding was the markedly elevated association between diabetic complications and depression. Microvascular complications (retinopathy, nephropathy, and neuropathy) were associated with a 17–18‐fold increase in the odds of depression, while macrovascular complications conferred a fourfold increase. Poor glycemic control was also strongly associated with depression, and achieving glycemic control was associated with an approximately 87%–88% reduction in depression odds. Finally, treatment regimen complexity (particularly insulin‐based therapies) was associated with greater depression severity, even when the binary presence of depression did not differ significantly by treatment type.

Given the cross‐sectional design, these associations should not be interpreted as causal. The term “associated with” is used throughout, and reverse causality (i.e., depression leading to poor glycemic control or complication development) remains plausible.

### 4.1. Comparison With Existing Literature

The 68% prevalence of depression in our T2DM cohort is higher than the 18%–25% typically reported in studies using structured clinical interviews [10]. However, it is consistent with studies using self‐report screening tools such as the PHQ‐9 or DASS‐21, which tend to capture broader psychological distress rather than only major depressive disorder [13]. For example, a study from East India using the DSM‐IV reported a prevalence of 29.2% [14], while a rural Indian study using the PHQ‐9 found only 6.2% [9]. This wide range reflects differences in assessment methods, cultural factors, and healthcare access.

To better contextualize our findings, Table 6 compares depression prevalence across studies, including a newly added study from Saudi Arabia [17], which reported a prevalence of 54% using the PHQ‐9 in a T2DM population [18]. The lower prevalence in rural India (6.2%) may be attributable to underreporting due to mental health stigma, different socioeconomic stressors, or the use of a different screening instrument [9].

**Table 6:** Comparison of depression prevalence in T2DM across studies.

Study	Population	Sample size	Assessment tool	Depression prevalence
Current study	Syrian (urban)	451	HDRS/DASS‐21	68.4%–68.9%
Alzahrani et al. [15]	Saudi Arabia	385	PHQ‐9	54.0%
Rural India study [9]	Rural India	420	PHQ‐9	6.2%
UK Biobank study [16]	UK nationwide	13,706	Hospital records/PHQ‐2	21.4% (with ASCVD)
East India study [14]	Mixed	Not specified	DSM‐IV	29.2%

The strong association between microvascular complications and depression aligns with prospective research showing that depression predicts subsequent vascular complications in T2DM patients. A recent UK Biobank study found that major depressive disorder was associated with a 32% increased risk of heart failure, 17% increased risk of atherosclerotic cardiovascular disease, and 29% increased risk of microvascular complications among T2DM patients [16]. Our findings extend this literature by quantifying the cross‐sectional association from the opposite direction: The presence of microvascular complications is associated with a substantially higher depression burden.

The gradient relationship between treatment complexity and depression severity has been reported previously but remains underappreciated in clinical practice. Insulin therapy, particularly in combination with oral agents, often signifies longer disease duration and greater treatment burden, which may contribute to psychological distress [10]. Our finding that 75% of patients with moderate‐to‐severe depression (by HDRS) were on combination therapy highlights this association.

### 4.2. Potential Mechanisms

The association between T2DM and depression is likely mediated by multiple biological and psychological pathways.

#### 4.2.1. Inflammatory Mechanisms

Both T2DM and depression are characterized by chronic low‐grade inflammation. Elevated proinflammatory cytokines (IL‐1*β*, IL‐6, and TNF‐*α*) are observed in both conditions and can cross the blood–brain barrier, affecting neurotransmitter metabolism (particularly serotonin and dopamine), neuroendocrine function, and neural plasticity [5, 19]. These inflammatory processes may be particularly relevant to anhedonic symptoms, which are common in diabetes and associated with poorer glycemic control [10].

#### 4.2.2. Neurodegeneration and Synaptic Impairment

Emerging evidence suggests that diabetic microvascular disease may directly affect brain regions involved in mood regulation. Chronic hyperglycemia and oxidative stress contribute to hippocampal synaptic dysfunction and reduced neuroplasticity. A recent study demonstrated that restoring hippocampal synaptic plasticity ameliorated depression‐like behaviors in diabetic animal models [20]. Similarly, the ESR2–miR‐10a‐5p–BDNF axis has been implicated in prefrontal cortex synaptic dysregulation contributing to depression‐like states [21]. While these findings are preclinical, they provide plausible mechanisms linking diabetic microvascular burden to mood disorders.

#### 4.2.3. Vascular and Endothelial Dysfunction

Microvascular complications in diabetes are associated with endothelial dysfunction, which may extend to the cerebral microvasculature. Reduced cerebral blood flow and blood–brain barrier disruption have been documented in both diabetes and depression [15]. A study on vascular growth factor synergies in endothelial and smooth muscle cells [17] and a clinical report on drug‐induced thrombotic microangiopathy as a microvascular injury model [22] offer comparative perspectives on how microvascular integrity affects tissue function, though their direct applicability to depression in T2DM requires further investigation.

#### 4.2.4. Psychosocial Pathways

The concept of diabetes‐related distress provides a complementary framework. The daily burdens of self‐management (blood glucose monitoring, medication adherence, and dietary restrictions), concerns about complications, and frustration with treatment regimens can lead to distress that manifests as depressive symptoms without necessarily meeting criteria for major depressive disorder [10]. This may explain why complication status and treatment complexity were such strong predictors in our study—these factors directly increase disease burden and reduce quality of life.

#### 4.2.5. Genetic and Bidirectional Causality

Mendelian randomization studies provide evidence for a bidirectional causal relationship between T2DM and depression. A recent study demonstrated that genetic liability to major depressive disorder was associated with 1.14 times higher odds of T2DM, while genetic liability to T2DM was associated with 1.02 times higher odds of major depressive disorder [19]. These genetic findings suggest shared biological pathways beyond the psychological impact of diabetes management, potentially involving insulin resistance, body fat distribution, and glycemic pathways.

### 4.3. Clinical Implications

Our findings have several implications for clinical practice.

First, the high prevalence of depression among T2DM patients—particularly those with complications, poor glycemic control, or complex treatment regimens—supports the implementation of routine depression screening in diabetes clinics. The consistent results across both HDRS and DASS in our study suggest that either clinician‐administered or self‐report tools can be effective. Culturally adapted instruments may be necessary in diverse populations [9].

Second, the strong association between treatment complexity and depression severity highlights the need for integrated care models that address both physiological and psychological aspects of diabetes. When intensifying treatment regimens (particularly when initiating insulin), clinicians should anticipate potential psychological impacts and provide additional support. Collaborative care models involving endocrinologists, psychiatrists, and diabetes educators have demonstrated effectiveness in improving both metabolic and mental health outcomes [10, 19].

Third, the strong association between microvascular complications and depression (OR ~ 17.5) suggests that preventing these complications may also be associated with lower depression risk. This supports intensive management of glycemic control and cardiovascular risk factors from diagnosis onward. Similarly, the protective association between glycemic control and depression (87%–88% reduction in odds) highlights the importance of supporting patients in achieving metabolic targets [16].

Based on these findings, we recommend the following clinical strategies:

1. *Routine depression screening* using validated tools during regular diabetes follow‐up visits, with increased vigilance for patients with complications or complex regimens.
2. *Integrated care models* that combine mental health support with diabetes management, particularly during treatment intensification.
3. *Prevention and management of microvascular complications* through intensive glycemic and cardiovascular risk factor control.
4. *Consideration of psychological factors* when making treatment decisions, balancing metabolic benefits against potential impacts on quality of life.

### 4.4. Limitations

Several limitations should be acknowledged.

#### 4.4.1. Cross‐Sectional Design

The most important limitation is the cross‐sectional design, which prevents the determination of causal relationships. While we describe associations, reverse causality (where depression contributes to complication development through poor self‐management) is equally plausible. Longitudinal studies are needed to establish temporal ordering.

#### 4.4.2. Sample Characteristics

The demographic characteristics of our sample—predominantly urban, employed, and married—may limit generalizability to rural populations, unemployed individuals, or those with lower social support. Convenience sampling for controls (hospital staff and visitors) may have introduced selection bias.

#### 4.4.3. Depression Assessment

Although we used two validated instruments, we did not employ structured clinical interviews (e.g., SCID), which represent the gold standard for major depressive disorder diagnosis. The high prevalence we observed may include individuals with subsyndromal depression or diabetes‐related distress rather than major depressive disorder. Self‐report bias (e.g., social desirability and recall bias) may have influenced DASS‐21 responses.

#### 4.4.4. Control Group Confirmation

We screened controls using FPG only, not an OGTT. Consequently, some controls with impaired glucose tolerance or early diabetes may have been misclassified. This limitation would bias results toward the null (i.e., underestimate the true association), so our effect sizes may be conservative.

#### 4.4.5. Glycemic Control Definition

The use of fasting blood glucose as a fallback when HbA1c was unavailable may overestimate glycemic control in some patients, as FBS reflects a single time point rather than chronic exposure.

#### 4.4.6. Small Reference Group for Microvascular Complications

Only 61 T2DM patients had no microvascular complications, resulting in wide CIs for the ORs (17.89; 95% CI [8.67–36.97]). This may have led to overestimation of the true effect size.

#### 4.4.7. Unmeasured Confounders

We did not assess medication effects (some antihyperglycemic agents may have mood effects), socioeconomic status beyond education, or social support networks, all of which could influence depression risk.

### 4.5. Future Directions

Future research should address these limitations through longitudinal designs that can establish temporal relationships and clarify causation. Studies examining intervention strategies targeted at high‐risk subgroups (e.g., those with complications or complex regimens) are needed to determine whether improving mental health can also improve diabetes outcomes. Mechanistic studies exploring the role of specific inflammatory markers, metabolic pathways, and genetic factors in the T2DM‐depression relationship could identify novel treatment targets [16, 19]. Finally, cultural adaptations of assessment tools and interventions are necessary, as depression prevalence and manifestations vary across populations. The notably lower prevalence found in rural Indian studies compared to our research highlights the importance of contextual factors [9].

## 5. Conclusion

This case‐control study provides evidence that the association between T2DM and depression is strongly modified by the clinical burden of the disease. Individuals with T2DM had approximately three times the odds of depression compared to nondiabetic controls. However, this risk was markedly amplified by the presence of diabetic complications: Microvascular complications were associated with a 17–18‐fold increase in depression odds. Achieving glycemic control was associated with an approximately 88% reduction in depression odds, and complex insulin‐based regimens were associated with greater depression severity.

These findings refine the understanding of the T2DM‐depression relationship from a simple comorbidity to a condition in which disease progression and treatment burden are key associated factors. The clinical implications support routine depression screening in diabetes care, particularly for high‐risk patients, and underscore that intensive diabetes management to prevent complications may also reduce depression risk.

## Author Contributions

**Mariam Wehbi:** conceptualization, data curation, investigation, methodology, writing – original draft, writing – review and editing. **Ahmad Al-Bitar:** conceptualization, data curation, formal analysis, investigation, methodology, writing – original draft, writing – review and editing. **Zaynab Alourfi:** methodology, supervision, writing – review and editing. **Younes Kabalan:** methodology, supervision, writing – review and editing.

## Funding

No funding was received for this manuscript.

## Disclosure

All authors have read and approved the final version of the manuscript. Ahmad Al‐Bitar (corresponding author) had full access to all of the data in this study and takes complete responsibility for the integrity of the data and the accuracy of the data analysis. All authors critically reviewed the manuscript for important intellectual content; all authors gave approval of the version to be submitted; all authors agreed to be accountable for all aspects of the work.

## Ethics Statement

This study received approval from the Biomedical Research Ethics Committee (BMREC) at Damascus University.

## Consent

All participants were informed about the study protocol and provided written informed consent.

## Conflicts of Interest

The authors declare no conflicts of interest.

## Data Availability Statement

The authors confirm that the data supporting the findings of this study are available within the article and its supporting information. Anonymized participant data may be requested from the corresponding author (Ahmad Al Bitar, ahmad.albitar@damascusuniversity.edu.sy) for academic purposes, subject to institutional ethical approval.

## Supporting information


**Supporting Information** Additional supporting information can be found online in the Supporting Information section. presents the adjusted association between Type 2 diabetes mellitus (T2DM) and depression using binary logistic regression controlling for age, sex, and educational level. Depression was assessed with two instruments: the DASS‐21 and the HDRS. After adjustment, T2DM diagnosis was associated with approximately threefold higher odds of depression (aOR ≈ 3.01 − 3.11), with strong statistical significance (p < 0.001).
