# Supplementary material for: Depression and Its Association With Microvascular and Macrovascular Complications in Type 2 Diabetes
Source: J Diabetes Res. 2026 May 11;2026:2234488. doi: 10.1155/jdr/2234488 (PMC13159080; doi:10.1155/jdr/2234488)
Supplement: Supplementary file 1 — Supporting Information Additional supporting information can be found online in the Supporting Information section. presents the adjusted association between Type 2 diabetes mellitus (T2DM) and depression using binary logistic regression controlling for age, sex, and educational level. Depression was assessed with two instruments: the DASS‐21 and the HDRS. After adjustment, T2DM diagnosis was associated with approximately threefold higher odds of depression (aOR ≈ 3.01 − 3.11), with strong statistical significance (p < 0.001). [file JDR-2026-2234488-s001.docx]

**Supplementary Table S1**

**Adjusted association between Type 2 Diabetes Mellitus and depression (binary logistic regression including age, sex, and education)**

| Outcome Measure | Predictor | Adjusted Odds Ratio (aOR) | 95% Confidence Interval | p-value |
| --- | --- | --- | --- | --- |
| Depression (DASS-21) | T2DM Diagnosis | 3.01 | 2.01 – 4.51 | <0.001 |
| Depression (HDRS) | T2DM Diagnosis | 3.11 | 2.08 – 4.65 | <0.001 |

**Covariates included in the model:** Age (continuous), sex (male/female), educational attainment (illiterate, primary, preparatory, secondary, university).
